# Supplementary material for: Biomarkers in Adult-Type Diffuse Gliomas: Elevated Levels of Circulating Vesicular Heat Shock Protein 70 Serve as a Biomarker in Grade 4 Glioblastoma and Increase NK Cell Frequencies in Grade 3 Glioma
Source: Biomedicines. 2023 Dec 7;11(12):3235. doi: 10.3390/biomedicines11123235 (PMC10741018; doi:10.3390/biomedicines11123235)
Supplement: Supplementary file 1 [file biomedicines-11-03235-s001.zip › biomedicines-2613933-supplementary.pdf]

Supplementary Figure S1

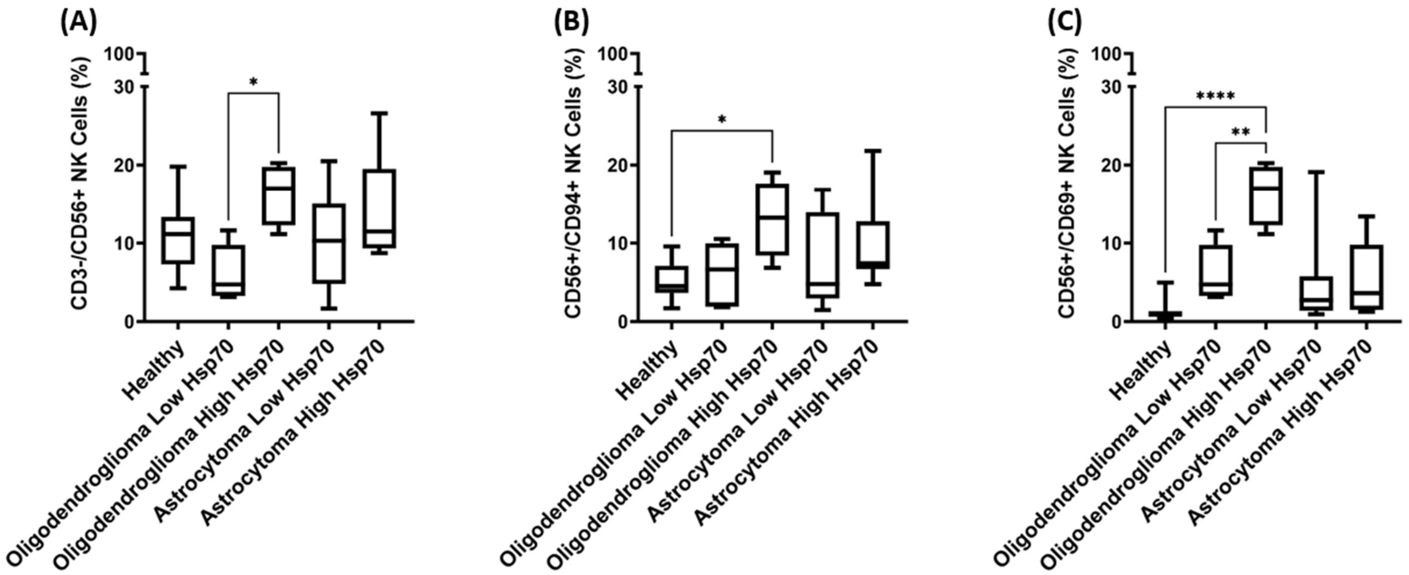

Supplementary Figure S1. Correlation of low and high Hsp70 values in the circulation and the proportion of different NK cell subpopulations in patients with oligodendroglioma ($Hsp70^{low}$ n=5; $Hsp70^{high}$ n=4) and astrocytoma ($Hsp70^{low}$ n=7; $Hsp70^{high}$ n=6) compared to a healthy control cohort (n=16). (A) CD3-/CD56+ NK cells in oligodendroglioma and astrocytoma patients with high ($Hsp70^{high}$) and low ($Hsp70^{low}$) Hsp70 plasma levels. (B) CD56+/CD94+ NK cells in oligodendroglioma and astrocytoma patients with high ($Hsp70^{high}$) and low ($Hsp70^{low}$) Hsp70 plasma levels. (C) CD56+/CD69+ NK cells in oligodendroglioma and astrocytoma patients with high ($Hsp70^{high}$) and low ($Hsp70^{low}$) Hsp70 plasma levels. Statistically significant differences *p<0.05, **p<0.01, ****p<0.0001.

Supplementary Figure S2

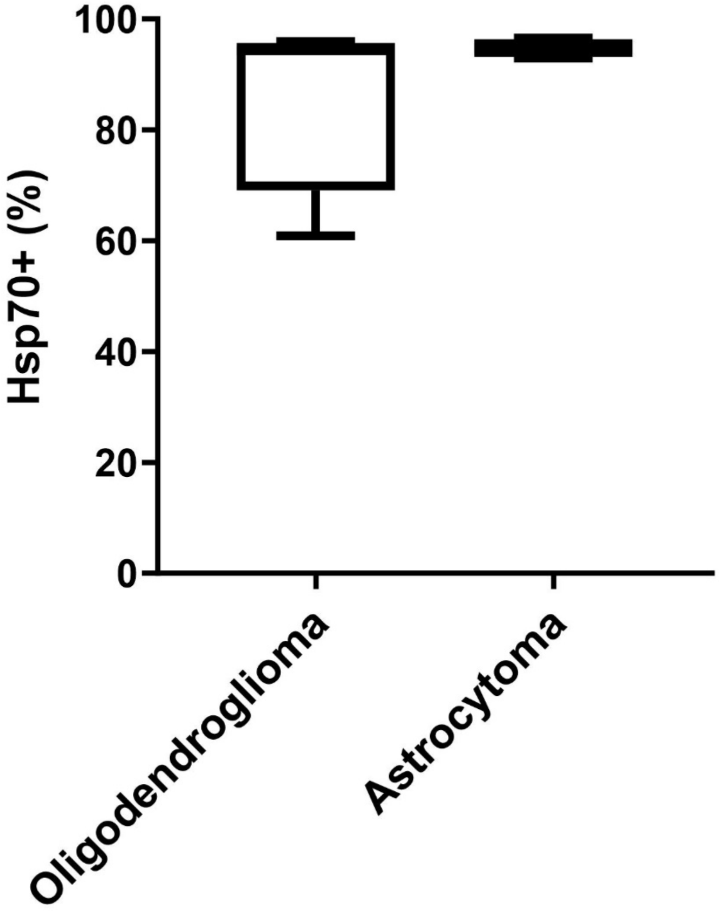

Supplementary Figure S2. The percentages of mHsp70 expressing cells in grade 3 oligodendroglioma (n=4) and grade 3 astrocytoma (n=3). No statistically significant differences.

Supplementary Figure S3

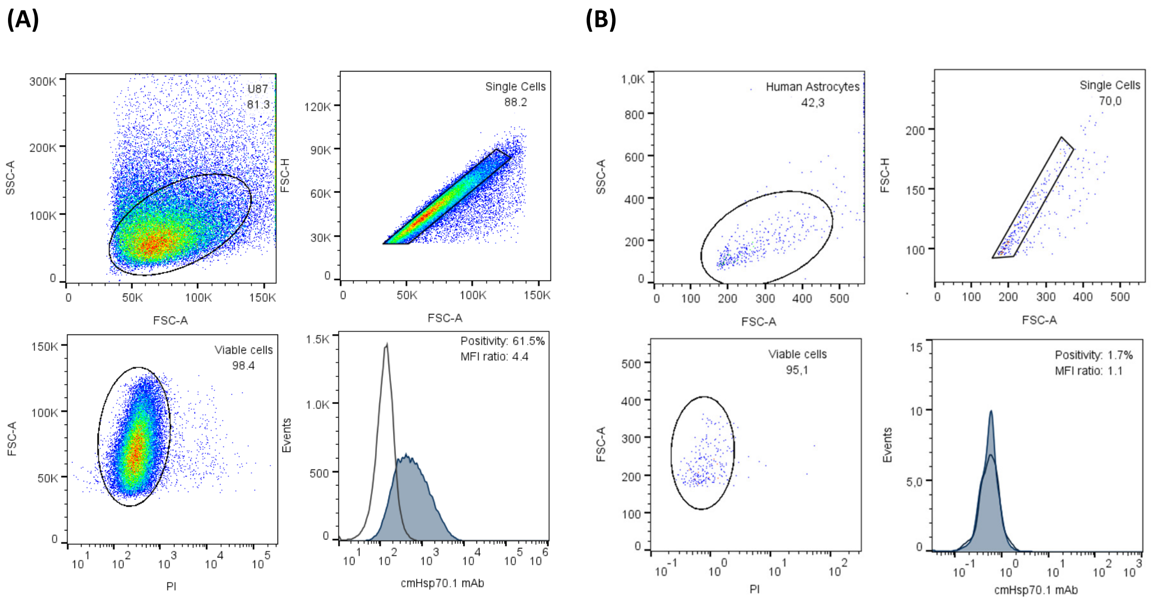

Supplementary Figure S3. Gating strategy and representative histograms of the percentage of mHsp70 expressing cells in cultured U87MG glioblastoma cells (A) and primary human astrocytes (B). The data represent one representative example of 3 independent experiments.
